# The association of breastfeeding with cognitive development and educational achievement in sub-Saharan Africa: A systematic review

**DOI:** 10.7189/jogh.12.04071

**Published:** 2022-09-03

**Authors:** Shamsudeen Mohammed, Laura L Oakley, Milly Marston, Judith R Glynn, Clara Calvert

**Affiliations:** 1 Department of Non-communicable Disease Epidemiology, Faculty of Epidemiology and Population Health, London School of Hygiene & Tropical Medicine, London, UK; 2 Centre for Fertility and Health, Norwegian Institute of Public Health, Oslo, Norway; 3 Department of Population Health, Faculty of Epidemiology and Population Health, London School of Hygiene & Tropical Medicine, London, UK; 4 Department of Infectious Disease Epidemiology, Faculty of Epidemiology and Population Health, London School of Hygiene & Tropical Medicine, London, UK; 5 Centre for Global Health, Usher Institute, University of Edinburgh, Edinburgh, UK

## Abstract

**Background:**

Systematic reviews and meta-analyses of studies mainly from high-income countries suggest that breastfeeding improves cognitive function and educational achievement. However, these associations may be a manifestation of who breastfeeds in these settings rather than an actual effect of breastfeeding. We investigated the association of breastfeeding with cognitive development and educational achievements in sub-Saharan Africa, where breastfeeding is the norm, and socioeconomic status is not strongly correlated with ever breastfeeding.

**Methods:**

We searched Medline, Embase, PsycINFO, Cochrane Central Register of Controlled Trials (CENTRAL), and Africa-Wide Information in January 2021 for studies that assessed the cognitive and educational benefits of breastfeeding in children and adolescents in sub-Saharan Africa. Two reviewers independently screened, extracted, and critically appraised the included studies.

**Results:**

After reviewing 5552 abstracts and 151 full-text articles, seventeen studies on cognitive development and two on educational achievements met our predefined inclusion criteria. The included studies were from ten sub-Saharan African countries and published between 2013 and 2021, with sample sizes ranging from 54 to 6573. Most of the studies (n = 14) were prospective cohort studies, but only nine collected data on breastfeeding prospectively. The studies differed in analytic approaches and cognitive and educational achievements measurements. Of the 17 studies on cognitive development, only four adjusted sufficiently for key confounders. None of these four studies found an overall association between breastfeeding and cognitive development in children or adolescents in sub-Saharan Africa. The two studies on education measured achievements based on the highest grade of school attained, 12 or more years of education, or grade repetition at age 7-11 years. Both studies adjusted for a range of sociodemographic factors and found no evidence that children exclusively breastfed or breastfed for a longer duration have a better educational outcome than sub-optimally breastfed children.

**Conclusions:**

The current evidence from sub-Saharan Africa is limited but does not corroborate previous findings that breastfeeding is associated with improved cognitive development and educational achievement.

**Registration:**

This study is registered with PROSPERO, CRD42021236009.

In 1929, Hoefer and Hardy reported a link between breastfeeding and cognitive development in their study of 7- to 13-year-olds in the United States [1], triggering a wave of studies investigating the association further. Since then, a number of systematic reviews and meta-analyses have evaluated and summarised the available evidence on the topic, with most of the reviews suggesting improved cognitive development and better educational outcomes in breastfed children [2-4]. For example, a meta-analysis commissioned by the World Health Organisation in 2007 found that breastfed children score 4.9 points higher on intelligence tests than non-breastfed children [3]. This meta-analysis was updated in 2013 [4] and again in 2015 [2], with results showing that breastfeeding was associated with 3.5 and 3.4 higher points in intelligence test scores, respectively.

There has been extensive debate on whether the association of breastfeeding with cognitive development and educational achievement is likely to be causal. It has been suggested that breastfed children have a higher cognitive function and educational achievement than non-breastfed children because of the high level of long-chain polyunsaturated fatty acids (LCPUFA) in breastmilk [5-7]. Nevertheless, in many settings, parents who breastfeed are likely to be different from parents who do not breastfeed (eg, with respect to socioeconomic status and maternal IQ) [8], and therefore any observed association with breastfeeding could be due to parental/family characteristics rather than a causal association with breastfeeding.

If breastfeeding does improve cognitive development and educational achievement, then we would expect to find evidence for this association across a range of different contexts; however, a review published in 2013 compared results from high-income and low- and middle-income countries and concluded that the 13 studies from low- and middle-income countries (LMICs) were more likely to report no effect of breastfeeding on cognitive development than the 71 studies from high-income countries [9]. Like other systematic reviews and meta-analyses exploring the association between breastfeeding and cognitive development or educational achievement [2,10], this review did not identify any studies from sub-Saharan Africa for inclusion [9].

Most of the research on breastfeeding in sub-Saharan Africa has been focused on the protective effects of breastfeeding on morbidity and mortality because of the high level of childhood infectious diseases in this region. Due to the lack of reviews on this topic focusing on sub-Saharan Africa, the cognitive and educational benefits of breastfeeding in the region are unclear. Language, childcare practices, and culture influence cognitive development, so it will be misleading to generalise reviews of studies from high-income countries to the sub-Saharan African context. We aimed to systematically review empirical evidence on the association of breastfeeding with cognitive development and educational achievement in children and adolescents in Sub-Saharan Africa.

## METHODS

### Protocol and registration

The protocol for the systematic review was registered with PROSPERO [11]. The Preferred Reporting Items for Systematic Reviews and Meta-Analyses (PRISMA) guided the structure and reporting of the review [12].

### Eligibility criteria

To be eligible for inclusion, studies had to meet the following criteria:

#### Participants

Studies were included if participants were children or adolescents aged 0-18 years in sub-Saharan Africa. Sub-Saharan Africa was defined as the 46 countries of Africa that lie south of the Sahara. For studies that investigated participants of all ages or analysed data from multiple countries, we included and extracted only the findings that met the eligibility criteria.

#### Exposure

Duration of breastfeeding and breastfeeding pattern appropriate to age were the exposures assessed. We excluded studies where breastfeeding information was collected five years or more after birth, as the length of recall is a potential source of bias [13]. We specified *a priori* to include studies regardless of confounder adjustment. However, for the narrative synthesis of results, we focused on studies that adjusted for maternal education or measures of socioeconomic status. Because of the strong correlation of breastfeeding with maternal education and measures of socioeconomic status, estimates from studies that do not adjust for these potential confounders can be misleading or biased.

#### Outcome measures

Studies had to report educational achievement or cognitive development as an outcome. Educational achievement was defined as the highest level of education attended or completed or how children and adolescents accomplished learning goals (eg, performance on a test). Cognitive development was defined as how children and adolescents think, process knowledge, solve problems, and develop skills. Included studies had to use validated age-appropriate methods to measure cognitive development. We excluded studies that solely assessed social and emotional-behavioural functioning as outcomes.

#### Type of study

Prospective and retrospective observational studies (cohort studies, case-control studies, and cross-sectional studies) and trials were considered for inclusion. There were no restrictions on language or publication date. Conference papers, reviews, and qualitative studies were excluded.

### Data sources and search strategy

A comprehensive literature search was conducted in Medline, Embase, PsycINFO, Cochrane Central Register of Controlled Trials (CENTRAL), and Africa-Wide Information electronic databases for relevant peer-reviewed articles from the databases’ inception. A search strategy was developed based on the review’s four main concepts: breastfeeding, educational achievement, cognitive development, and sub-Saharan Africa. First, a brief search was conducted in each database to determine the free-text terms, keywords, and synonyms used in the thesaurus to describe the main concepts. A full search strategy was then constructed with guidance from previous reviews [2,14-17] using a combination of subject headings and a wide range of free-text terms and synonyms for the main concepts. The search was refined through an iterative process and in consultation with a specialist Librarian (see Table S1 in the **Online Supplementary Document** for the full search strategy). The database searches were conducted in January 2021. Reference lists and citations of included studies and existing reviews and meta-analyses were searched (backward and forward reference searching). The backward and forward reference searching was conducted in the Web of Science Core Collection and Google Scholar in July 2021.

### Screening of studies and data extraction

Articles retrieved from the database searches were imported to Mendeley citation manager, and duplicates were removed. A second deduplication was carried out in a more sensitive system [18], the Rayyan systematic review manager [19], to ensure complete deduplication of the search results. Authors SM and CC initially screened 20% of the titles and abstracts against the eligibility criteria for relevance. SM screened the remaining titles and abstracts, and CC checked them for consistency. SM obtained and read the full text of the articles kept after the initial screening to exclude those that did not meet the predefined inclusion criteria. For studies with no full text available online, SM attempted contact with the authors. CC reviewed decisions on 25% of the full-text articles screened using the same criteria.

Data extraction was performed by SM and CC using a data extraction form developed in Microsoft Excel. The form contained fields for study setting and design, data collection methods, study population and sample size, age at recruitment and follow-up period, breastfeeding measurement, assessment of education, cognitive outcomes and covariates, and a summary of findings before and after adjustment. The extraction form was piloted on four articles, and based on the pre-test results, modifications were made to the form. We attempted to contact authors of studies for more information where necessary. Disagreements between reviewers were settled through discussions.

### Critical appraisal of studies

The criteria used to judge the methodological quality of the included studies were adapted from the Joanna Briggs Institute’s (JBI) critical appraisal tools for analytical cross-sectional studies [20], cohort studies [21], and randomised controlled trials (RCTs) [22]. SM conducted the quality appraisal, and CC checked a sample for consistency. The presence or absence of a criterion was denoted with “low risk of bias” or “high risk of bias”, respectively, and “unclear” was used where authors did not provide sufficient information to judge a criterion.

### Data synthesis

We decided, *a priori,* to conduct a meta-analysis if at least two studies were homogeneous with respect to definition, classification, or measurement of breastfeeding and cognitive development or educational achievements and used comparable analytic approaches [11]. However, after reviewing the studies, a meta-analysis was deemed inappropriate given the heterogeneity of breastfeeding classification, outcome measurements, and analytic techniques. Instead, we conducted a narrative synthesis focusing on studies that controlled for maternal education or measures of socioeconomic status and other important confounders in the design or analysis. Effect estimates and 95% confidence intervals from the included studies were extracted and presented in tables. In studies where cognitive development or educational achievement were measured as continuous variables, we showed the mean differences of these outcomes by breastfeeding groups. Odds ratios were reported for studies where the outcomes were recorded as dichotomous variables. Because the estimates were derived using different methodologies and analytic approaches, we did not pool or combine them to produce a single overall effect estimate; each study was presented separately in the tables.

### Ethics approval

Ethical approval was not required for this study.

## RESULTS

**Figure 1:**
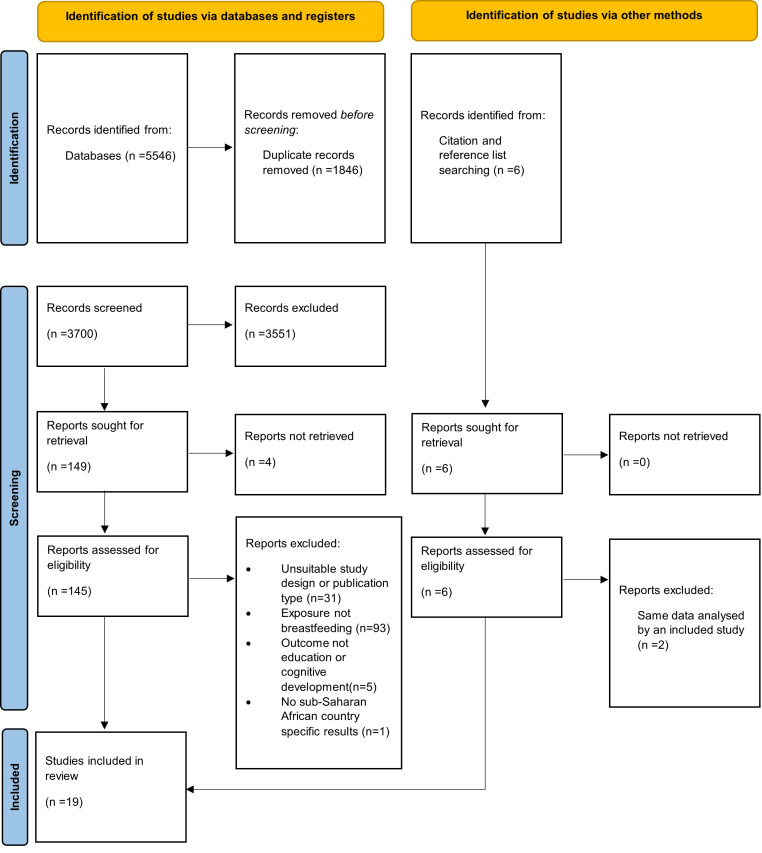
PRISMA flow diagram of the study selection process.

A flowchart of the study selection process is presented in **Figure 1**. Overall, 5546 potentially relevant articles were found in five databases. Six additional articles were identified through hand-searching. After removing duplicates (n = 1846), and screening titles and abstracts, 151 articles remained. Full texts of the 151 articles were reviewed, and 19 articles were eligible for inclusion. Of the 19 included articles, 17 were on cognitive development and two on educational achievements.

### Critical appraisal of included studies

Each study was assessed for the possibility of selection bias, measurement bias, and confounding (Table S2 in the **Online Supplementary Document**). For most of the studies, the criteria for inclusion were clearly defined, and children or adolescents from the same population were compared. However, in 27% of the studies, there was a substantial loss to follow-up (over 20%), and 44% of the studies did not provide sufficient information to decide on the extent of loss to follow-up. In 21% of the studies, breastfeeding information was collected retrospectively. More than half of the studies (10/19) did not provide sufficient information to judge if breastfeeding was measured similarly in the groups. All 17 studies on cognitive development used validated tools to assess cognition. Most studies (13/19) did not adjust sufficiently for potential confounders.

### Breastfeeding and cognitive development in sub-Saharan Africa

Of the 17 studies on cognitive development, 12 were prospective cohort studies [23-34], four were cross-sectional studies [35-38], and one was a cluster RCT [39] (**Table 1** and Table S3 in the **Online Supplementary Document**). These studies were published between 2013 and 2021, with the majority (88%) published in the last five years. There was high variability in sample sizes, ranging from 54 to 6573. The studies were conducted across ten sub-Saharan African countries, but most were done in South Africa [23,27,29,30,31,34,36]. Three studies were multicentred, pooling data from Uganda and Burkina Faso [39], Uganda and Malawi [24], and Ghana and Malawi [33].

**Table 1:** Characteristics and findings of studies on cognitive development and educational achievements that adjusted sufficiently for relevant confounders

No	Author(s) name, year of publication	Setting	Study design	Study dates	Aim of study	Description of study population	Measurement of breastfeeding	Breastfeeding groups compared	Assessment of cognitive and educational outcome
**A. Cognitive development**										
1	Rochat et al., 2016 [23]	South Africa	Prospective cohort study	2012-2014	To investigate the association between exclusive breastfeeding, HIV exposure, and other early and current life factors and later cognitive development, executive function, and emotional behavioural development in children aged 7 to 11 y.	906 HIV-negative primary school-aged children born to HIV-positive and HIV-negative mothers enrolled in an intervention cohort study supporting them to practice exclusive breastfeeding for the first 180 d of life in a rural area of South Africa.	The total number of days in the first six months that the child received only breastmilk was collected and then divided by 30 into months, irrespective of whether the days were sequential.	Exclusive breastfeeding for 2-5 mo and 6 mo vs 0-1 mo.	At age 7-11 y, the Kaufman Assessment Battery for Children (KABC-II) was used to assess cognitive development.
2	Namazzi et al., 2019 [38]	Uganda	Cross-sectional	2017	To establish the prevalence and factors associated with neurodevelopmental disability among infants in Eastern Uganda.	487 infants born between January and March 2017 in the Iganga/Mayuge Health Demographic Surveillance Site (HDSS) in Eastern Uganda.	Unclear	Exclusive breastfeeding for six months vs non-exclusive breastfeeding	Neurodevelopment was assessed in December 2017 using the Malawi Developmental Assessment Tool (MDAT).
3	Tumwine et al., 2018 [39]	Uganda and Burkina Faso	Community-based cluster-randomised trial	2013-2015	To assess the effects of exclusive breastfeeding peer counselling in the first six months of life on cognitive abilities, social-emotional development, school performance and linear growth among 5–8 y old children in Uganda and Burkina Faso.	1083 children residing in selected clusters in rural Banfora, Southwest Burkina Faso, and from the Mbale District, Eastern Uganda.	Unclear	Exclusive breastfeeding status at 12 weeks	At 5-8 y, the Kaufman Assessment Battery for Children, second edition (KABC-II), the Test of Variables of Attention (T.O.V.A.1) and the Children's Category Test (CCT 1) were used to assess child development.
4	Le Roux et al., 2018 [30]	South Africa	Prospective birth cohort	2013-2017	To assess neurodevelopment of breastfed HIV-exposed uninfected (HEU) and breastfed HIV-unexposed children in the context of universal maternal antiretroviral therapy (ART)	521 participants, of which 215 were HEU and 306 HIV-unexposed children born to women who initiated ART in pregnancy in a peri-urban community and breastfed their children.	Information on breastfeeding duration was determined from maternal self-report of infant feeding practices at 6 weeks; 3, 6, 9 and 12 mo, with the last visit taken as the last day of breastfeeding.	Breastfeeding duration in months	At age 11–18 mo, the Bayley Scales of Infant and Toddler Development, Third Edition (BSID-III), was used to assess child development.
**B. Educational achievement**										
1	Horta et al., 2013 [40]	South Africa	Prospective cohort study	1990-2009	To assess the association between early feeding practices and long-term school achievement.	2225 babies born to predominantly poor black women residing in an area of Johannesburg.	Information on infant feeding practices was collected from mothers when the cohort members were 3, 6, 12, and 24 mo old on whether they were ever breastfed, if they were still breastfeeding, and for those who had stopped, when they stopped, and when complementary foods were introduced.	Ever breastfed vs never breastfed. Breastfeeding ≤1 mo vs >1-3 mo, >3-6 mo, >6-12 mo >12-18 mo >18-24 mo >24 mo	The highest school grade attained and completion of ≥12 y of schooling.
2	Mitchell et al., 2016 [41]	South Africa	Prospective cohort study	2001-2014	To investigate early life factors associated with earliest repeated grade and explore child characteristics and parental reports of reasons for failure among early repeaters.	894 HIV-negative children born and residing in northern KwaZulu-Natal whose mothers were alive and had participated in an intervention to support exclusive breastfeeding.	Exclusive breastfeeding was estimated as the number of days a child received only breast milk and no other fluids or solids. This was divided into months.	Exclusive breastfeeding 0–1-mo vs 2-5 mo, and 6 mo	At age 7-11 y, mothers or caretakers were asked about their children's schooling history and any repeated grades.

y – year, mo – months

Exclusive breastfeeding, duration of breastfeeding, or ever breastfed were the three main breastfeeding exposures. 17 different psychometric tools were used to measure cognitive development, with five studies using multiple tools. The most frequently used psychometric tool was the Bayley Scales of Infant and Toddler Development (BSID). Most of the studies (n = 12) only included participants younger than three years (range six months to 11 years).

11 of the cognitive development studies did not adjust for any confounders [24-29,33-37], and two studies adjusted for some confounders but not maternal education or family socioeconomic status [31,32] (Table S3 and S4 Table in the **Online Supplementary Document**). Only four studies controlled for maternal education or measures of family socioeconomic status [23,30,38,39], with one of these adjusting for maternal intelligence [23].

None of the four studies that adjusted for maternal education or family socioeconomic status found evidence of an association between breastfeeding and cognitive development in the overall sample (**Table 2**). Namazzi et al. assessed cognitive development in nine to 12 months olds (n = 487) using the Malawi Developmental Assessment Tool (MDAT) [38]. Exclusive breastfeeding at six months was the exposure, and adjustment was made for maternal education, maternal age, birth weight, post-neonatal complications, number of childhood illnesses and other confounders in the analysis. There was no evidence in the adjusted analysis that exclusive breastfeeding at six months improved children's cognitive development (**Table 2**). In Le Roux et al.'s study of 521 breastfed HIV-exposed uninfected and HIV-unexposed children in South Africa, the BSID-III was used to assess cognitive development aged 11-18 months [30]. After adjusting for maternal education, HIV status, alcohol use and intimate partner violence (IPV), infant gestational age at birth, and birth size, the mean differences of the three subscales of BSID-III showed no evidence of improved cognitive function in those breastfed for a longer duration (**Table 2**).

**Table 2:** Estimates of the effect of breastfeeding on cognitive development in sub-Saharan Africa from studies adjusted sufficiently for relevant confounders

				Odds ratios (95% CI)																								
**Study 1: Rochat et al., 2016** [23]*																												
	**Sequential**						**Planning**							**Learning**				**Simultaneous**						**Riddle**				
		**Unadjusted odds ratio (95% CI)**			**Adjusted odds ratio (95% CI)**			**Unadjusted odds ratio (95% CI)**			**Adjusted odds ratio (95% CI)**		**Unadjusted odds ratio (95% CI)**			**Adjusted odds ratio (95% CI)**			**Unadjusted odds ratio (95% CI)**			**Adjusted odds ratio (95% CI)**		**Unadjusted odds ratio (95% CI)**		**Adjusted odds ratio (95% CI)**		
**Exclusive breastfeeding (overall sample)**																												
0-1 mo	1.00			1.00			1.00			1.00				1.00		1.00		1.00			1.00			1.00			1.00	
2-5 mo	1.09 (0.7 to 1.7)			1.27 (0.8 to 2.1)			0.93 (0.6 to 1.5)			0.96 (0.6 to 1.6)				0.93 (0.6 to 1.5)		1.16 (0.7 to 1.9)		1.02 (0.6 to 1.6)			1.34 (0.8 to 2.2)			0.76 (0.5 to 1.2)			1.07 (0.6 to 1.8)	
6 mo	1.01 (0.7 to 1.6)			1.23 (0.8 to 2.0)			0.75 (0.5 to 1.1)			0.80 (0.5 to 1.3)				1.04 (0.7 to 1.6)		1.29 (0.8 to 2.1)		0.94 (0.6 to 1.4)			1.29 (0.8 to 2.1)			0.77 (0.5 to 1.2)			1.18 (0.7 to 1.9)	
										
**Study 2: Namazzi et al., 2019** [38]†																												
	**Neuro-developmental disability**																											
	**Unadjusted odds ratio (95% CI)**			**Adjusted odds ratio (95% CI)**			-			-				-		-		-			-			-			-	
**Exclusive breastfeeding for six months**																												
Yes	1.00			1.00			-			-				-		-		-			-			-			-	
No	1.37 (0.70 to 2.67)			0.62 (0.30 to 1.27)			-			-				-		-		-			-			-			-	
	**Mean difference (95% confidence interval)**																											
**Study 3: Tumwine et al., 2018** [39]‡																												
	**General cognition**						**Working memory**							**Attention**				**Inhibition**						**Cognitive flexibility**				
			**Unadjusted mean difference (95% CI)**			**Adjusted mean difference (95% CI)**			**Unadjusted mean difference (95% CI)**			**Adjusted mean difference (95% CI)**			**Unadjusted mean difference (95% CI)**		**Adjusted mean difference (95% CI)**			**Unadjusted mean difference (95% CI)**			**Adjusted mean difference (95% CI)**		**Unadjusted mean difference (95% CI)**			**Adjusted mean difference (95% CI)**
Exclusive breastfeeding promotion intervention	0.08 (-0.13 to 0.29)			-0.07 (-0.30 to 0.15)			-0.01 (-0.32 to 0.29)			-0.07 (-0.29 to 0.16)				0.12 (-0.02 to 0.26)		0.11 (-0.13 to 0.35)		0.03 (-0.17 to 0.23)			-0.05 (-0.28 to 0.19)			-0.07 (-0.21 to 0.07)			0.02 (-0.27 to 0.30)	
**Study 4: Le Roux et al., 2018** [30]§																												
	**Cognitive development**						**Motor development**							**Language development**				-			-			-			-	
			**Unadjusted mean difference (95% CI)**			**Adjusted mean difference (95% CI)**			**Unadjusted mean difference (95% CI)**			**Adjusted mean difference (95% CI)**			**Unadjusted mean difference (95% CI)**		**Adjusted mean difference (95% CI)**			-			-		-			-
Breastfeeding duration (months)	0.11 (-0.11 to 0.34)			0.09 (-0.13 to 0.31)			0.19 (-0.04 to 0.41)			0.15 (-0.08 to 0.37)				0.02 (-0.22 to 0.26)		0.01 (-0.24 to 0.25)		-			-			-			-	

CI – confidence interval

*Adjusted for child sex, child age, mother's age at birth, maternal IQ, mother's education at birth, birthweight, birth order, mother's HIV status, residence, income provider, owning fridge, perception of wealth, crèche, HOME assessment score, maternal mental health, and parenting stress.

†Adjusted for father's occupation, maternal age, maternal education, tetanus toxoid to the mother, marital status, mother parity, ANC attendance, place of delivery, birth weight, cried at birth, post-neonatal complications, and number childhood illnesses.

‡Adjusted for socioeconomic status, electricity in-home, duration in kindergarten and cluster.

§Maternal HIV, education, alcohol use and IPV; infant gestational age at birth, and birth size.

In Rochat et al.’s prospective study of 906 HIV-negative primary school-aged children in South Africa [23], cognitive development was assessed at 7-11 years using the Kaufman Assessment Battery for Children (KABC-II). After controlling for the mother's education at birth, ownership of fridge, perception of wealth, maternal intelligence, and other sociodemographic confounders, there was no evidence that a longer duration of exclusive breastfeeding was associated with improved cognitive development (**Table 2**). However, there was weak evidence in the sex-stratified analysis that boys exclusively breastfed for longer were two times more likely to score above the mean for learning ability sub-scale than those breastfed for less than a month (Table S5 in the **Online Supplementary Document**). It was unclear whether the subgroup analysis was prespecified, and there was no benefit in the other four domains of development or for girls.

In Tumwine et al.'s cluster RCT of exclusive breastfeeding peer counselling in Burkina Faso and Uganda (n = 1083), cognitive development was assessed at age 5-7 using the KABC-II, Test of Variables of Attention (T.O.V.A) and the children's category test (CCT) [39]. In the intention to treat analysis, there was no evidence that being randomised to exclusive breastfeeding peer counselling was associated with improved cognitive development on any of the psychometric tools used (**Table 2**). Electricity in-home, SES, and kindergarten attendance were imbalanced at baseline and were controlled in the analysis. In a secondary analysis ignoring the randomisation, there was evidence of improved inhibition among children exclusively breastfed at 12 weeks. It was unclear whether the secondary analysis was prespecified, and there was no evidence of improved performance on the other four cognitive measures (Table S5 in the **Online Supplementary Document**).

### Breastfeeding and educational achievement in sub-Saharan Africa

Two prospective cohort studies assessed the effect of breastfeeding on educational achievements in South Africa. The breastfeeding exposures and the measures of educational achievements differed in the two studies (**Table 1**).

In Horta et al.’s study of 2225 children (average age of 17.7 years) born to predominantly poor black women in Johannesburg [40], the highest grade of schooling attained and completion of 12 or more years of education were the measures of educational achievement. The exposures were ever breastfed and the duration of breastfeeding. After adjusting for socioeconomic status at birth, maternal schooling, skin colour, maternal age, smoking during pregnancy, birthweight, age at follow-up, and sex, there was no evidence that either breastfeeding indicator was associated with better grade progression or completion of schooling than never breastfeeding or breastfeeding for a month or less (**Table 3**).

**Table 3:** Estimates of the effect of breastfeeding on educational achievement in sub-Saharan Africa

Study 1: Horta et al., 2013 [40]*				
	**Highest grade achieved at school**		**Completed at least 12 y of schooling**	
	**Unadjusted mean difference (95% CI)**	**Adjusted mean difference (95% CI)**	**Unadjusted prevalence ratio (95% CI)**	**Adjusted prevalence ratio (95% CI)**
**Ever breastfeed**				
No	0.0	0.0	1.00	1.00
Yes	0.02 (-0.26 to 0.29)	-0.08 (-0.41 to 0.25)	0.97 (0.84 to 1.13)	0.95 (0.79 to 1.14)
**Duration of any breastfeeding (in months)**				
≤1.00	0.0	0.0	1.00	1.00
>1-3	-0.06 (-0.32 to 0.19)	-0.23 (-0.52 to 0.05)	0.92 (0.80 to 1.05)	0.83 (0.70 to 0.98)
>3-6	-0.10 (-0.38 to 0.18)	-0.10 (-0.41 to 0.21)	0.94 (0.81 to 1.09)	0.93 (0.78 to 1.10)
>6-12	0.04 (-0.22 to 0.30)	-0.03 (-0.31 to 0.26)	0.98 (0.85 to 1.12)	0.97 (0.83 to 1.12)
>12-18	-0.02 (-0.29 to 0.25)	-0.08 (-0.37 to 0.21)	0.97 (0.84 to 1.11)	0.94 (0.80 to 1.09)
>18-24	0.16 (-0.13 to 0.45)	0.09 (-0.21 to 0.40)	1.01 (0.87 to 1.17)	1.04 (0.89 to 1.21)
>24	-0.12 (-0.37 to 0.12)	-0.02 (-0.29 to 0.24)	0.89 (0.78 to 1.02)	0.97 (0.84 to 1.12)
				
**Study 2: Mitchell et al., 2016** [41]†				
	**Grade repetition**			
	**Unadjusted odds ratio (95% CI)**	**Adjusted odds ratio (95% CI)**	**-**	**-**
**Exclusive breastfeeding for six months (Overall sample; n = 842)**				
0-1 mo	1.00	1.00	-	-
2-5 mo	0.77 (0.49 to 1.22)	0.76 (0.45 to 1.28)	-	-
6 mo	0.70 (0.46 to 1.07)	0.64 (0.39 to 1.06)	-	-

mo – months

*Adjusted for socioeconomic status at birth, maternal schooling, skin colour, maternal age, smoking during pregnancy, birthweight, subject's age at follow-up, and sex.

†Adjusted for maternal age, maternal education, residence, main income, and fridge ownership (all measured at birth) and child age, child sex, birth order, birth weight, HIV exposure.

Mitchell et al. re-enrolled 894 7-11-year-olds in northern KwaZulu-Natal [41]. The outcome was grade repetition at age 7-11 years and the exposure was exclusive breastfeeding in the first six months. The analysis controlled for maternal age, maternal education, residence, main income, and fridge ownership (all measured at birth) and child age, sex, birth order, birth weight, and HIV exposure. There was no evidence that a longer duration of exclusive breastfeeding was associated with favourable school progression (**Table 3**). Likewise, in sex-stratified subgroup analysis, there was no evidence of an association between exclusive breastfeeding and grade repetition in girls or boys (Table S6 in the **Online Supplementary Document**).

## DISCUSSION

In this systematic review, we identified 17 studies assessing the effects of breastfeeding on cognitive development and two studies on breastfeeding and educational achievement in children and adolescents in sub-Saharan Africa. However, only four studies on cognitive development and the two studies on educational achievement adjusted sufficiently for relevant confounders. Overall, we found no evidence that breastfeeding is associated with improved cognitive development or higher educational achievement in children or adolescents in sub-Saharan Africa from the studies sufficiently adjusted for confounders. Two studies found some association with cognitive development in subgroup analysis [23,39]. However, it is unclear whether these subgroup analyses were pre-specified.

In contrast to our findings, many previous systematic reviews and meta-analyses have reported an association between breastfeeding and cognitive development. A 1999 meta-analysis of 11 studies found that breastfed children scored 3.2 points higher on cognitive function tests than formula-fed children. The effect persisted until adolescence, showing a dose-response relationship [42]. A systematic review of three studies in 2007 reported higher educational achievement in breastfed children in late adolescence and young adulthood [3]. In a 2019 review of 73 studies, the majority of the studies demonstrated a positive effect of breastfeeding on cognitive development and educational achievements [10]. Similarly, in a 2015 meta-analysis of 17 studies, Horta et al., found that breastfed children scored 3.4 points higher on intelligence tests than non-breastfed children [2].

Based on these reviews and results from a cluster randomised breastfeeding intervention trial in Belarus, Horta et al., suggested that the association is causal [2]. However, none of these reviews that reported a beneficial effect of breastfeeding on cognitive development included a study from sub-Saharan Africa. Moreover, the Belarusian cluster randomised trial only found evidence of association on two measures of verbal intelligence (vocabulary (aβ = +4.9, 95% CI = +0.4 to +9.3), similarities (aβ = +4.6, 95% CI = +0.2 to +9.0), and overall verbal IQ (aβ = +7.5, 95% CI = +0.8 to +14.3)) of the Wechsler Abbreviated Scales of Intelligence (WASI) [43]. There was no significant difference between the experimental and control groups on the other two subtests of WASI that measured performance (nonverbal) intelligence (block designs (aβ = +1.9, 95% CI = -1.7 to +5.5); matrices (aβ = +1.8, 95% CI = -1.9 to +5.5); and overall performance IQ (aβ = +2.9, 95% CI = -3.3 to +9.1)). In addition, the trial did not find evidence of a difference in intelligence on full-scale IQ of WASI (aβ = +5.9, 95% CI = -1.0 to 12.8) [43].

Researchers have suggested that the presence of arachidonic acid (ARA) and docosahexaenoic acid (DHA) in breastmilk are responsible for the improved cognitive advantage in breastfed children [44] as these two long-chain polyunsaturated fatty acids (LCPUFAs) are critical for brain development [45]. However, the results of this systematic review cast doubt on this potential causal pathway, as we would expect to see similar associations in breastfed children from diverse populations, including African children. In fact, studies have shown that breastfeeding mothers in Africa and Asia have the highest level of breastmilk DHA worldwide [46]. Furthermore, a meta-analysis of 12 RCTs to examine the efficacy of LCPUFA supplementation of infant formula on cognitive development found no evidence that LCPUFA supplementation of formula improves early cognitive function [47]. It is possible that the constituents of breastmilk are not responsible for the observed positive associations in previous reviews, but rather reflect the methodological challenges in measuring the association between breastfeeding and both cognitive functioning and education status.

Most of the individual studies included in the previous reviews were from high-income countries (HICs), where exclusive breastfeeding and longer breastfeeding duration are more common in mothers with higher education and family income [8]. As higher SES is also associated with improved cognitive development and educational achievements in these settings [48-50], it is possible that the social advantage of breastfed children confounds the beneficial effect of breastfeeding reported in these reviews and the benefits are not true of breastfeeding. Although the reviews and meta-analyses were carefully designed with strict inclusion criteria to minimise the effect of confounding, residual confounding from family socioeconomic status is still possible.

Reviews that included studies from low- and middle-income countries (LMICs), where maternal education and family socioeconomic status do not greatly influence breastfeeding [8], reported null or inconclusive results. For example, when the association between breastfeeding and educational achievement was assessed in five birth cohorts from LMICS (Brazil, India, Guatemala, the Philippines, and South Africa), investigators found no evidence of an association between breastfeeding and educational achievements [40]. A review of 84 studies in 2013 found that studies from LMICs were about two times more likely to report no cognitive benefits of breastfeeding than those from HICs. The investigators concluded that the positive effect seen in HICs was probably due to residual confounding from maternal intelligence and family socioeconomic status [9].

It is also possible that the psychometric tools used to measure cognitive development accounted for the absence of evidence of an association between breastfeeding and cognitive development in sub-Saharan Africa. Most studies in this review assessed cognitive development with psychometric tools developed and validated in HICs. Despite evidence of cross-cultural differences in developmental trajectories due to factors other than intellectual abilities [51-53], when these tools assess cognitive development in sub-Saharan Africa, children’s performance is compared to the norm-referenced scores established among children in HICs [54-56]. In a study to validate the BSID-III in Malawi, investigators found that using the US-based norms misclassified the neurological development of about 25%-36% of Malawian children across the subscales of the tool [55]. Misclassification and, consequently, misleading results are likely in the studies that used these tools without adapting them to the local context. Nevertheless, the studies that used the Malawi Development Assessment Tool and Kilifi Developmental Inventory found no cognitive benefit from breastfeeding [26,28,33,38]. Future studies on cognitive development should use culturally sensitive psychometric tools or adapt and validate existing tools to the local context and, where possible, adopt standardised approaches to defining exposure, outcome, and the analysis to facilitate comparison between studies.

We found wide variation in the breastfeeding exposure indicators and psychometric tools used to assess cognitive development. Different analytic approaches precluded a meta-analysis even where studies used the same breastfeeding indicator. Of the studies on cognitive development, 11 did not adjust for any potential confounders, probably because most studies were not designed to investigate the cognitive benefits of breastfeeding. In addition, two studies that adjusted for confounders failed to adjust for some important known confounders, including maternal education and measures of SES. The studies included in the present review were conducted in only ten of the 46 sub-Saharan African countries. Both studies on educational outcomes were from South Africa, demonstrating the lack of studies on this topic.

## CONCLUSIONS

The current evidence in sub-Saharan Africa does not corroborate the findings elsewhere that breastfeeding is associated with improved cognitive development and educational achievements in children and adolescents. However, this conclusion was based on a small number of studies, and the measurements of cognitive development and educational achievements and the analytic approaches used varied considerably across the studies. We echo the World Health Organisation’s recommendation on breastfeeding infants from birth to age two years and beyond since there is considerable evidence elsewhere [57] that breastfeeding protects against gastrointestinal infection in children. Whether it also benefits cognitive and educational outcomes remains unclear. There is a need for high-quality research on the educational benefits of breastfeeding in sub-Saharan Africa since this has far-reaching implications for the future of children and adolescents.

## Additional material


Online Supplementary Document
