# Supplementary material for: The association of breastfeeding with cognitive development and educational achievement in sub-Saharan Africa: A systematic review
Source: J Glob Health. 2022 Sep 3;12:04071. doi: 10.7189/jogh.12.04071 (PMC9441109; doi:10.7189/jogh.12.04071)
Supplement: Online Supplementary Document [file jogh-12-04071-s001.pdf]

ONLINE SUPPLEMENTARY DOCUMENT

Title: The association of breastfeeding with cognitive development and educational achievement in sub-Saharan Africa: a systematic review

Authors: Shamsudeen Mohammed[1*], Laura L Oakley[1,2], Milly Marston[3], Judith R Glynn[4], and Clara Calvert[5]

Table S 1: Criteria for database searches in Medline, Embase, PsycINFO, Cochrane Central Register of Controlled Trials (CENTRAL), and Africa-Wide Information

Medline		
	**Search strategy**	**Results**
1	exp breast feeding/ or exp lactation/ or exp Bottle Feeding/ or exp infant nutritional physiological phenomena/ or exp Infant Formula/ or exp Milk, Human/ or exp Lactation/ or weaning/	108568
2	(Breast* adj3 (fe* or milk)).ti,ab,kw.	43487
3	((Infant or child) adj3 (fe* or nutrition or formula or milk)).ti,ab,kw.	30289
4	(formula adj3 (fe* or milk)).ti,ab,kw.	7424
5	((Bottle or cup) adj fe*).ti,ab,kw.	2385
6	((human or artificial) adj3 (milk or lactation)).ti,ab,kw.	12122
7	1 or 2 or 3 or 4 or 5 or 6	149514
8	exp cognition/ or exp intelligence/ or exp Intelligence Tests/	280236
9	((cognit* or intell*) adj2 (develop* or outcom* or function* or process* or abilit* or tes* or quotient or perform* or capab*)).ti,ab,kw.	125099
10	8 or 9	356232
11	exp academic performance/ or exp academic success/ or exp Educational Measurement/ or exp Educational Status/	199466

12	((educat* or academic* or school* or exam* or test*) adj3 (perform* or achiev* or attain* or grade* or level* or success* or status* or outcome* or result* or mark* or score* or progress* or atten* or assess* or evalua* or measur*)).ti,ab,kw.	750027
13	11 or 12	906082
14	10 or 13	1209396
15	exp "africa south of the sahara"/	212602
16	((sub-Sahara* or east* or west* or southern or central) adj2 Africa*).ti,ab,kw.	43331
17	(((Angola* or Benin* or Botswana or Burkina Faso or Burundi* or Cameroon* or Republique du Cameroun or Cape Verde* or Cabo Verde* or Central African Republic or Republique centrafricaine or Chad* or Comoros or Congo* or Democratic Republic of the Congo or Cote d'Ivoire or Ivory Coast or Djibouti* or Eritrea* or Ethiopia* or Gabon* or Republique gabonaise or Gambia* or Ghana* or Equatorial Guinea or Guinea* or Republique de Guinee or Guinea Bissau or Republica da Guine-Bissau or Kenya* or Lesotho or Liberia* or Madagascar or Malawi* or Mali* or Mauritania* or Mauriti* or Mayotte or Mozambi* or Namibia* or Republic of the Niger or Republique du Niger or Niger* or Nigeria* or Rwanda* or Democratic Republic of Sao Tome) and Principe) or Republica Democratica de Sao Tome e Principe or Senegal* or Republique du Senegal or Seychell* or Sierra Leone* or Somalia* or South Africa* or Sudan* or Swaziland or eSwatini or Togo* or Uganda* or United Republic of Tanzania or Zambia* or Zimbabwe*).ti,ab,kw.	390069
18	15 or 16 or 17	423932
19	7 and 14 and 18	830

Embase

	Search strategy	Results
1	exp breast feeding/ or exp lactation/ or exp Bottle Feeding/ or exp infant nutritional physiological phenomena/ or exp Infant Formula/ or exp Milk, Human/ or exp Lactation/ or weaning/	170201
2	(Breast* adj3 (fe* or milk)).ti,ab,kw.	58029
3	((Infant or child) adj3 (fe* or nutrition or formula or milk)).ti,ab,kw.	45733
4	(formula adj3 (fe* or milk)).ti,ab,kw.	11515
5	((Bottle or cup) adj fe*).ti,ab,kw.	3180
6	((human or artificial) adj3 (milk or lactation)).ti,ab,kw.	17289
7	1 or 2 or 3 or 4 or 5 or 6	226710

8	exp cognition/ or exp cognitive development/ or exp cognitive function test/ or exp intelligence/ or exp intellect/ or exp intelligence quotient/ or exp psychomotor development/	2476232
9	((cognit* or intell*) adj2 (develop* or outcom* or function* or process* or abilit* or tes* or quotient or perform* or capab*)).ti,ab,kw.	218212
10	8 or 9	2528653
11	exp academic performance/ or exp academic success/ or exp Educational Measurement/ or exp Educational Status/	1606956
12	((educat* or academic* or school* or exam* or test*) adj3 (perform* or achiev* or attain* or grade* or level* or success* or status* or outcome* or result* or mark* or score* or progress* or atten* or assess* or evalua* or measur*)).ti,ab,kw.	1456018
13	11 or 12	2831819
14	10 or 13	4838250
15	exp "africa south of the sahara"/	365622
16	((sub-Saharan or east or west or southern or central) adj2 Africa*).ti,ab,kw.	64371
17	(((Angola* or Benin* or Botswana or Burkina Faso or Burundi* or Cameroon* or Republique du Cameroun or Cape Verde* or Cabo Verde* or Central African Republic or Republique centrafricaine or Chad* or Comoros or Congo* or Democratic Republic of the Congo or Cote d'Ivoire or Ivory Coast or Djibouti* or Eritrea* or Ethiopia* or Gabon* or Republique gabonaise or Gambia* or Ghana* or Equatorial Guinea or Guinea* or Republique de Guinee or Guinea Bissau or Republica da Guine-Bissau or Kenya* or Lesotho or Liberia* or Madagascar or Malawi* or Mali* or Mauritania* or Mauriti* or Mayotte or Mozambi* or Namibia* or Republic of the Niger or Republique du Niger or Niger* or Nigeria* or Rwanda* or Democratic Republic of Sao Tome) and Principe) or Republica Democratica de Sao Tome e Principe or Senegal* or Republique du Senegal or Seychell* or Sierra Leone* or Somalia* or South Africa* or Sudan* or Swaziland or eSwatini or Togo* or Uganda* or United Republic of Tanzania or Zambia* or Zimbabwe*).ti,ab,kw.	526181
18	15 or 16 or 17	647807
19	7 and 14 and 18	2644
	**Pshyinfo**	
	**Search strategy**	**Results**
1	exp breast feeding/ or exp lactation/ or exp Bottle Feeding/ or weaning/	5788
2	(Breast* adj3 (fe* or milk)).ti,ab.	3168

3	((Infant or child) adj3 (fe* or nutrition or formula or milk)).ti,ab.	8234
4	(formula adj3 (fe* or milk)).ti,ab.	536
5	((Bottle or cup) adj fe*).ti,ab.	422
6	((human or artificial) adj3 (milk or lactation)).ti,ab.	261
7	1 or 2 or 3 or 4 or 5 or 6	14815
8	exp Cognitive Development/ or exp cognition/ or exp cognitions/ or exp cognitive ability/ or exp cognitive assessment/ or exp executive function/ or exp intelligence/ or exp intelligence measures/ or exp intelligence quotient/ or exp Intellectual Development/	390464
9	((cognit* or intell*) adj2 (develop* or outcom* or functio* or process* or abilit* or tes* or achieve* or quotient or perform* or capab*)).ti,ab.	162596
10	8 or 9	471134
11	exp academic achievement/ or exp Academic Aptitude/ or exp Educational Attainment Level/ or exp educational measurement/ or exp education/	486454
12	((educat* or academic* or school* or exam* or test*) adj3 (perform* or achiev* or attain* or grad* or level* or success* or status* or outcome* or result* or mark* or score* or progress* or atten* or assess* or evalua* or measur*)).ti,ab.	418273
13	11 or 12	769568
14	10 or 13	1130189
15	((sub-Saharan or east* or west* or south* or central) adj2 Africa*).ti,ab.	20596
16	(((Angola* or Benin* or Botswana or Burkina Faso or Burundi* or Cameroon* or Republique du Cameroun or Cape Verde* or Cabo Verde* or Central African Republic or Republique centrafricaine or Chad* or Comoros or Congo* or Democratic Republic of the Congo or Cote d'Ivoire or Ivory Coast or Djibouti* or Eritrea* or Ethiopia* or Gabon* or Republique gabonaise or Gambia* or Ghana* or Equatorial Guinea or Guinea* or Republique de Guinee or Guinea Bissau or Republica da Guine-Bissau or Kenya* or Lesotho or Liberia* or Madagascar or Malawi* or Mali* or Mauritania* or Mauriti* or Mayotte or Mozambi* or Namibia* or Republic of the Niger or Republique du Niger or Niger* or Nigeria* or Rwanda* or Democratic Republic of Sao Tome) and Principe) or Republica Democratica de Sao Tome e Principe or Senegal* or Republique du Senegal or Seychell* or Sierra Leone* or Somalia* or South Africa* or Sudan* or Swaziland or eSwatini or Togo* or Uganda* or United Republic of Tanzania or Zambia* or Zimbabwe*).ti,ab.	43737

17	15 or 16	49554
18	7 and 14 and 17	90

**Cochrane library**		
**ID**	**Search strategy**	**Hits**
#1	MeSH descriptor: [Breast Feeding] explode all trees	1857
#2	MeSH descriptor: [Infant Nutritional Physiological Phenomena] explode all trees	2792
#3	MeSH descriptor: [Bottle Feeding] explode all trees	218
#4	MeSH descriptor: [Weaning] explode all trees	136
#5	MeSH descriptor: [Infant Formula] explode all trees	610
#6	MeSH descriptor: [Milk, Human] explode all trees	999
#7	((Breast* or formula or bottle or cup) NEAR/3 (fe* or milk))	10763
#8	((human or artificial) NEAR/2 (milk or lactation))	3073
#9	((human or artificial) NEAR/2 (milk or lactation))	3073
#10	#1 or #2 or #3 or #4 or #5 or #6 or #7 or #8 or #9	12202
#11	MeSH descriptor: [Cognition] explode all trees	10149
#12	MeSH descriptor: [Intelligence] explode all trees	5775
#13	(cognit* or intell*) NEAR/3 (develop* or outcom* or function* or process* or abilit* or tes* or achieve* or quotient or perform* or capab*)	33011
#14	#11 or #12 or #13	43475
#15	MeSH descriptor: [Academic Performance] explode all trees	74
#16	MeSH descriptor: [Academic Success] explode all trees	48
#17	MeSH descriptor: [Education] explode all trees	32544
#18	MeSH descriptor: [Educational Measurement] explode all trees	4673
#19	MeSH descriptor: [Educational Status] explode all trees	1444
#20	((educat* or academic* or school* or exam* or test*) NEAR/3 (perform* or achiev* or attain* or grade* or level* or success* or status* or outcome* or result* or mark* or score* or progress* or atten* or assess* or evalua* or measur*))	147955
#21	#15 or #16 or #17 or #18 or #19 or #20	170168

#22	#14 or #21	198480
#23	MeSH descriptor: [Africa South of the Sahara] explode all trees	6328
#24	(*sahara* or sub-Sahara* or east* or west* or south* or central) NEAR/3 (Africa*)	10608
#25	Angola* or Benin* or Botswana or “Burkina Faso” or Burundi* or Cameroon* or “Republique du Cameroun” or “Cape Verde*” or “Cabo Verde*” or “Central African Republic” or “Republique centrafricaine” or Chad* or Comoros or Congo* or “Democratic Republic of the Congo” or “Cote d'Ivoire” or “Ivory Coast” or Djibouti* or Eritrea* or Ethiopia* or Gabon* or “Republique gabonaise” or Gambia* or Ghana* or “Equatorial Guinea” or Guinea* or “Republique de Guinee” or “Guinea Bissau” or “Republica da Guine-Bissau” or Kenya* or Lesotho or Liberia* or Madagascar or Malawi* or Mali* or Mauritania* or Mauriti* or Mayotte or Mozambi* or Namibia* or “Republic of the Niger” or “Republique du Niger” or Niger* or Nigeria* or Rwanda* or “Democratic Republic of Sao Tome and Principe” or “Republica Democratica de Sao Tome e Principe” or Senegal* or “Republique du Senegal” or Seychell* or “Sierra Leone*” or Somalia* or “South Africa*” or Sudan* or Swaziland or eSwatini or Togo* or Uganda* or “United Republic of Tanzania” or Zambia* or Zimbabwe*	22178
#26	#23 or #24 or #25	24537
#27	#10 and #22 and #26	305
**Africa-Wide Information**		
	**Search strategy**	**Results**
S11	S4 AND S7 AND S10	(1,677)
S10	S8 OR S9	(2,915,364<br>)
S9	Angola* or Benin* or Botswana or “Burkina Faso” or Burundi* or Cameroon* or “Republique du Cameroun” or “Cape Verde*” or “Cabo Verde*” or “Central African Republic” or “Republique centrafricaine” or Chad* or Comoros or Congo* or “Democratic Republic of the Congo” or “Cote d'Ivoire” or “Ivory Coast” or Djibouti* or Eritrea* or Ethiopia* or Gabon* or “Republique gabonaise” or Gambia* or Ghana* or “Equatorial Guinea” or Guinea* or “Republique de Guinee” or “Guinea Bissau” or “Republica da Guine-Bissau” or Kenya* or Lesotho or Liberia* or Madagascar or Malawi* or Mali* or Mauritania* or Mauriti* or Mayotte or Mozambi* or Namibia* or “Republic of the Niger” or “Republique du Niger” or Niger* or Nigeria* or Rwanda* or “Democratic Republic of Sao Tome and Principe” or “Republica Democratica de Sao Tome e Principe” or Senegal* or “Republique du Senegal” or Seychell* or “Sierra Leone*” or Somalia* or “South Africa*” or Sudan* or Swaziland or eSwatini or Togo* or Uganda* or “United Republic of Tanzania” or Zambia* or Zimbabwe*	(2,822,743<br>)
S8	(sub-Sahara* or subsahara* or east* or west* or south* or central) N3 (Africa*)	(2,048,410<br>)
S7	S5 OR S6	(107,065)

S6	((educat* or academic* or school* or exam* or test*) N3 (perform* or achiev* or attain* or grade* or level* or success* or status* or outcome* or result* or mark* or score* or progress* or atten* or assess* or evalua* or measur*))	(100,937)
S5	(cognit* or intell*) N3 (develop* or outcom* or function* or process* or abilit* or tes* or achieve* or quotient or perform* or capab*)	(8,318)
S4	S1 OR S2 OR S3	(21,059)
S3	((human or artificial) N3 (milk or lactation))	(1,302)
S2	((Infant or child) N3 (fe* or breast* or breast-fe* or nutrition or formula or milk))	(16,643)
S1	((Breast* or formula or bottle or cup) N2 (fe* or milk))	(7,237)

Table S 2: Results of risk of bias assessment of included studies

No.	Authors	Were the criteria for inclusion in the sample clearly defined?	Were the exposed and unexposed groups similar and recruited from the same population?	Was follow up complete for all participants?	Were breastfeeding data collected prospectively?	Was breastfeeding measured similarly to assign people to both exposed and unexposed groups?	Were educational achievement or cognitive development measured validly or reliably?	Were family income or maternal education controlled for in the design or analysis?	Were other confounding factors controlled for in the design or analysis?
**A. Cognitive development**									
1	Rochat et al., 2016(1)	Yes	Yes	≤20% LTFU	Yes	Yes	Yes	Yes	Yes, see table 2
2	Namazzi et al., 2019(2)	No	Yes	Not stated	Unclear	Unclear	Yes	Yes	Yes, see table 2
3	Tumwine et al., 2018(3)	Yes	Yes	Over 20% LTFU	Yes	Unclear	Yes	Yes	Yes, see table 2
4	Le Roux et al., 2018(4)	Yes	Yes	≤20% LTFU	No	Yes	Yes	Yes	Yes, see table 2
5	Madlala et al., 2020(5)	Yes	Yes	Over 20% LTFU	Unclear	Unclear	Yes	No	Yes, see appendix p 8
6	McDonald et al., 2013(6)	Yes	Yes	Unclear	Yes	Yes	Yes	No	Yes, see appendix p 8
7	Boivin et al., 2019(7)	Yes	Yes	≤20% LTFU	Yes	Unclear	Yes	No	No
8	Ngoma et al., 2014(8)	Yes	Yes	Not stated	Yes	Unclear	Yes	No	No
9	Strehlau et al., 2020(9)	Yes	Yes	Not stated	Unclear	Unclear	Yes	No	No
10	Eales et al., 2020(10)	Yes	Yes	Not stated	No	Unclear	Yes	No	No
11	Prado et al., 2017(11)	No	Yes	Not stated	Yes	Yes	Yes	No	No
12	Donald et al., 2019(12)	Yes	Yes	Over 20% LTFU	Yes	Yes	Yes	No	No
13	Chaudhury et al., 2021(13)	Yes	Yes	≤20% LTFU	Unclear	Unclear	Yes	No	No
14	Ekholuenetale et al., 2020(14)	No	Yes	N/A	No	Yes	Yes	No	No
15	Namazzi et al., 2020(15)	Yes	Yes	≤20% LTFU	Unclear	Unclear	Yes	No	No
16	White et al., 2020(16)	Yes	Yes	Over 20% LTFU	No	Yes	Yes	No	No
17	Sirajee et al., 2021(17)	Yes	Yes	Over 20% LTFU	Unclear	Unclear	Yes	No	No
**B. Educational achievements**									
1	Horta et al., 2013(18)	Yes	Yes	Not stated	Yes	Yes	Unclear	Yes	Yes, see table 3
2	Mitchell et al., 2015(19)	Yes	Yes	Not stated	Yes	Yes	Yes	Yes	Yes, see table 3
Total									
		3	0	5	4	0	0	13	11
		16	19	5	9	9	18	6	8
		0	0	8	6	10	1	0	0
Legend		Low risk of bias		High risk of bias		Unclear			

LTFU: loss to follow-up

Note: studies are organised based on the order in which they appeared in Table 1 and appendix page 8 (characteristics of studies included in the review).

Table S 3: Characteristics and findings of studies on cognitive development not adjusted sufficiently for maternal education or measures of socioeconomic status

No	Author(s) name, year of publication, study setting and design.	Aim of study	Description of study Population	Measurement of breastfeeding	Breastfeeding groups compared	Assessment of cognitive and educational outcome	Summary of findings before adjustment	Covariates and confounders adjusted for	Summary of findings after adjustment
5	Madlala et al., 2020(5)<br><br>**Setting:** South Africa<br><br>**Study design**: Prospective cohort study<br><br>**Study dates**: April 2015-May 2018	To examine the association of maternal and child factors with neurodevelopment at 12–24 months of age.	355 live singleton HEU children whose mothers initiated ART pre- or during pregnancy and were attending primary health care at Gugulethu Community Health Centre, a semi-urban area with a population predominantly made up of black African ethnic group with low socioeconomic status.	Unclear	Ever breastfed vs never breastfed.<br><br>Breastfeeding <6months vs breastfeeding ≥6months (restricted to ever breastfed children)	At age 12-24 months, the Ages and Stages Questionnaire (ASQ) was used to assess neurodevelopment.	There was no evidence of association between ever breastfed and neurodevelopm ent.<br><br>Breastfeeding for ≥6 months was associated with decreased odds of delayed neurodevelopm ent (specifically a measuring combining communication + problem-solving + personal-social)	Sex, size for gestational age, delivery gestational age, and weight-for-age at assessment	After adjustment, there was no evidence of association between ever breastfed and neurodevelopme nt.<br><br>Breastfeeding for ≥6 months was no longer associated with neurodevelopme nt after adjustment
6	McDonald et al., 2013(6)<br><br>**Setting**: Tanzania<br><br>**Study design**: Prospective cohort study<br><br>**Study dates**: unclear	To identify the maternal, socioeconomic, and child correlates of psychomotor and mental development in the first 18 months of life among Tanzanian infants born to HIV infected women.	311 children of mothers enrolled in a trial to examine the effect of multivitamin supplements on mother-to-child HIV-1 transmission and disease progression.	Information on breastfeeding practices was collected from mothers monthly for a minimum of 18 months from birth.	Duration of exclusive breastfeeding ≥3months vs <3months	At 6, 12, and 18 months of age, the Psychomotor Development Index (PDI) and Mental Development Index (MDI) of the Bayley Scales of Infant Development, 2nd edition (BSID-II) were used to assess child development.	There was no evidence of association between the duration of exclusive breastfeeding and PDI or MDI of the BSID-II.	Child age at cognitive assessment and parent study treatment regimen.	After adjustment, there was no evidence of association between duration of breastfeeding and PDI or MDI of the BSID-II.
7	Boivin et al., 2019(7)	To compare neurodevelopmental outcomes of	861 children: 405 HEU children whose mothers received antiretroviral	Information on breastfeeding practices were collected at 12	Breastfeeding at 12months vs No	At age 12, 24, and 48 months the Mullen Scales of Early Learning (MSEL) was	In Uganda, breastfeeding was associated with lower	Not available	Not available

	**Setting**: Uganda and Malawi<br><br>**Study design**: Prospective cohort study<br><br>**Study dates**: 2013-2014	antepartum and post-partum antiretroviral exposure in HIV-exposed uninfected (HEU) children with HIV-unexposed and uninfected (HUU) children aged 12, 24, 48, and 60 months.	prophylaxis during pregnancy and triple antiretroviral treatment or infant nevirapine during breastfeeding and 456 HUU in Blantyre and Kampala.	and 24 months from primary caregivers.	breastfeeding at 12 months	administered. At 48 and 60 months, the Kaufman Assessment Battery for Children, second edition (KABC-II), was administered to assess child development.	MSEL cognitive score in HEU children but with higher scores in HUU children. There was no evidence of association between breastfeeding and the KABC II index in either group.<br><br>In Malawi, there was no evidence of association between breastfeeding and MSEL score or KABC II index.		
8	Ngoma et al., 2014(8)<br><br>**Setting**: Zambia<br><br>**Study design**: Cross-sectional study<br><br>**Study dates**: 2011-2013	To determine whether there is a higher risk for cognitive or language delay among children exposed to ART in utero and through 1 year of breastfeeding, compared with children born to HIV-uninfected mothers	200 HEU children born to women enrolled in the Aluvia Study and living within a single district in Lusaka.	Unclear	Duration of breastfeeding in weeks	At age 15-36 months, the Capute Scales Clinical Adaptive Test/Clinical Linguistic and Auditory Milestone Scale (CAT/CLAMS) was used to assess child development.	There was no evidence of association between the duration of breastfeeding and odds of having a Capute Full-Scale Developmental Quotient (FSDQ) score less than 85.	Not available	Not available
9	Strehlau et al., 2020(9)<br><br>**Setting**: South Africa	To describe neurodevelopmental assessment results at 12 months of age from a cohort of HEU children from similar socioeconomic backgrounds.	70 HEU children with no congenital abnormalities or history of significant birth trauma delivered at the Rahima Moosa Mother and Child	Unclear	Exposure to breastmilk	At age 48 weeks the Bayley Scales of Infant and Toddler Development-3rd Edition (BSID-III) was used to assess child development.	There was no evidence of association between exposure to breastmilk and achieving a composite	Not available	Not available

	**Study design**: Prospective cohort study<br><br>**Study dates**: 2016-2018		Hospital, Johannesburg.				score of ≥110 using the Bayley Scales.		
10	Eales et al., 2020(10)<br><br>**Setting**: South Africa<br><br>**Study design**: Cross-sectional study<br><br>**Study dates**: Unclear	To determine the relationship between infant feeding characteristics and developmental outcomes	144 children attending a well-baby immunisation clinic in the Tshwane District, Gauteng province of South Africa.	Information on participants feeding history were collected from parent/caregiver's using a questionnaire.	unclear	At between 6-12 months, child development was assessed with the Parent's Evaluation of Developmental Status (PEDS) tools and Parent's Evaluation of Developmental Status – Developmental Milestones (PEDS-DM)	There was an association between ever breastfed, receipt of breastmilk, colostrum, and appropriate receptive language skills.<br><br>Longer breastfeeding duration was associated with appropriate expressive language skills.	Not available	Not available
11	Prado et al., 2017(11)<br><br>**Setting**: Ghana and Malawi<br><br>**Study design**: Prospective cohort study<br><br>**Study dates**: unclear	To identify the factors associated with 18-month language and motor development and the pathways through which these factors operate.	3083 children from two countries (Ghana and Malawi) who participated in the International Lipid-Based Nutrient Supplements (iLiNS) Project, which assigned participants to receive various doses and formulations of lipid-based nutrient supplements (LNS) or to control groups.	Information on child feeding practices was collected using a (24hour and/or 7-day) dietary recall questionnaire administered to mothers/caregivers at multiple time points from age 1 to 5 months.	Exclusive Breastfeeding for the first 6 months	At age 18 months, the Kilifi Developmental Inventory (KDI) and a 100-word vocabulary checklist were used to assess motor and language development in Ghana and Malawi. In Burkina Faso, the Developmental Milestones Checklist-II (DMC-II) was used.	There was no evidence of association between exclusive breastfeeding and language or motor development in both Ghana and Malawi.	Not available	Not available
12	Donald et al., 2019(12)	To investigate the risk and protective factors of early childhood	734 children residing in Paarl, a low-socioeconomic	Information on infant feeding practices was collected from mothers	Duration of exclusive breastfeeding	At age 24 months, the Bayley Scales of Infant and Toddler	There was no evidence of association	Not available	Not available

	**Setting**: South Africa<br><br>**Study design**: Prospective birth cohort<br><br>**Study dates**: 2012-2015	developmental outcomes and determine sex differences in the impact of such factors in a birth cohort.	community 60km away from Cape Town.	at birth, 6, 10, and 14 weeks, and at 6 and 9 months.	in first 6 months	Development (Third Edition) (BSID-III) was used to assess Child development.	between exclusive breastfeeding and child development		
13	Chaudhury et al., 2021(13)<br><br>**Setting**: Botswana<br><br>**Study design**: Prospective birth cohort<br><br>**Study dates**: 2010-2012	To determine if HIV-exposed uninfected (HEU) children had worse neurodevelopmental outcomes at 24 months compared with HIV-unexposed uninfected (HUU) children.	905 children of HIV-infected and uninfected mothers residing in Gaborone and Mochudi in Botswana	Unclear	Ever breastfed vs never breastfed	At age 22-29 months, the Bayley Scales of Infant and Toddler Development, Third Edition (Bayley-III) and the Development Milestones Checklist (DMC) was used to assess child development.	There was no evidence of association between ever breastfed and child development	Not available	Not available
14	Ekholuenetal e et al., 2020(14)<br><br>**Setting**: Benin<br><br>**Study design**: Cross-sectional<br><br>**Study dates**: 2017-18	To examine the impact of stunting on cognitive development of children under-5 years in Benin.	6573 children born within the 5 years prior to the 2017-18 Benin Demographic and Health Survey survey was used with a sample of 6573 children included from BDHS—2017/ 18 survey.	Information on infant breastfeeding practices was collected from mother's.	Duration of breastfeeding <12 months vs 12months or more.	Cognitive development was measured using a set of 8 items: (1) child can identify or name at least 10 letters of alphabet, (2) child can read at least four words, (3) child can identify or name at least 10 numbers, (4) child can pick up small object with two fingers, (5) child follows simple directions, (6) child can perform task independently, (7) child gets along well with other children or adults, and (8) child does not get distracted easily.	There was no evidence of association between breastfeeding and cognitive development.	Not available	Not available

15	Namazzi et al., 2020(15)<br><br>**Setting**: Uganda<br><br>**Study design**: Prospective cohort study<br><br>**Study dates**: May 2018 to May 2019	To determine the neurodevelopmental outcomes among preterm infants and identify any modifiable factors associated with neurodevelopmental disability.	398 children (187 born at term and 211 preterm) delivered at the Iganga hospital in Eastern Uganda between May and July 2018 and whose mothers were residents within a 20 km radius area of the hospital.	Unclear	Exclusive breastfeeding in first six months vs mixed feeding.	At age 6 to 8 months, the Malawi Developmental Assessment tool was used to assess neurodevelopment.	There was no evidence of association between exclusive breastfeeding and neurodevelopmental disability.	Not available	Not available
16	White et al., 2020(16)<br><br>**Setting**: South Africa<br><br>**Study design**: Prospective cohort study<br><br>**Study dates**: 2016 to 2017	To determine the effects of HIV on growth and immune- and neurodevelopment in infants in early life	54 children (22 HIV uninfected and 32 HIV infected) whose mothers delivered at the Kalafong Provincial Tertiary Hospital in Pretoria between June and December 2016.	At 8 to 16 weeks follow-up after birth, mothers reported whether they were, or had ever, exclusively breastfed their infants. If the infants were currently receiving formula, and the age at which formula was introduced.	Exclusive breastfeeding at follow-up	At 8 to 16 weeks postpartum, the Guide for Monitoring Child Development (GMCD) was used to assess neurodevelopment.	There was no evidence of association between exclusive breastfeeding and infant neurodevelopment.	Not available	Not available
17	Sirajee et al., 2021(17)<br><br>**Setting**: Uganda<br><br>**Study design**: Prospective cohort study<br><br>**Study dates**: 2016 to 2018	To determine the associations between prenatal and postnatal growth and subsequent neurodevelopment in Ugandan HEU infants	170 HIV-Exposed, Uninfected Infants delivered at the Jinja Regional Referral Hospital and Kambuga District Hospital in Uganda.	Unclear	Exclusive breastfeeding at 6weeks, 12 and 18 months, and breastfed within 1 hour after birth.	At age 12 to 18 months, the Malawi Development Assessment Tool (12 and 18 months) and the Colour Object Association Test (18 months) were used to assess child development.	There was no evidence of association between exclusive breastfeeding or breastfeeding within 1 hour, and infant neurodevelopment.	Not available	Not available

Table S 4: Estimates of the effect of breastfeeding on cognitive development in sub-Saharan Africa from studies not adjusted for maternal education or measures of socioeconomic status

**Study 5: Madlala et al., 2020**(5)*					-
	**Gross + Fine motor**		**Comm + ProbSolv + PerSocial**		-
	**Unadjusted odds ratio (95% CI)**	**Adjusted odds ratio (95% CI)**	**Unadjusted odds ratio (95% CI)**	**Adjusted odds ratio (95% CI)**	-
**Breastfeeding**					
Never	1.00	1.00	1.00	1.00	-
Ever	0.99 (0.46-2.14)	1.23 (0.49-3.09)	0.61 (0.25-1.49)	1.07 (0.35-3.26)	-
**Breastfeeding duration**					
< 6 months	1.00	-	1.00	-	-
≥ 6 months	0.72 (0.45-1.17)	-	0.50 (0.26-0.97)	-	-

**Study 6: McDonald et al., 2013**(6)†					
	**Psychomotor Development Index (PDI)**		**Mental Development Index (MDI)**		-
	**Unadjusted mean difference (95% CI)**	**Adjusted mean difference (95% CI)**	**Unadjusted mean difference (95% CI)**	**Adjusted mean difference (95% CI)**	-
**Duration of exclusive breastfeeding (months)**					
≥3	0.00	0.00	0.00	-	-
<3	2.32 (-0.54; 5.17)	1.04 (-1.50; 3.59)	1.16 (-0.92; 3.23)	-	-

**Study 7: Boivin et al., 2019**(7)					
	**Uganda**		**Malawi**		-
	**MSEL composite cognitive score**	**KABC mental processing index**	**MSEL composite cognitive score**	**KABC mental processing index**	-
	**Mean difference (SE), p-value**	**Mean difference (SE), p-value**	**Mean difference (SE), p-value**	**Mean difference (SE), p-value**	-
**HIV-exposed and uninfected children (PROMISE 1077-BF)**					
Breastfeeding at 12 months	- 4.38(1.54), 0.005	-2.16 (1.60), 0.18	-3.99 (2.64), 0.13	-7.39 (3.13), 0.02	-
No breastfeeding at 12 months	0.00	0.00	0.00	0.00	-
**HIV-unexposed and uninfected children (controls)**					
Breastfeeding at 12 months	5.96 (2.34), 0.0119	1.49 (2.76), 0.59	4.73 (9.32), 0.61	-6.82 (7.98), 0.39	-
No breastfeeding at 12 months	0.00	0.00	0.00	0.00	-

**Study 8: Ngoma et al., 2014**(8)					
	**HIV-exposed uninfected**	**Control**	**P-value**	**P-value for univariable regression**	-
	**Mean (SD)**	**Mean (SD)**			
Duration of breastfeeding (weeks)	50.0 (9.0)	71.6 (24.6)	<0.001	0.12	-

**Study 9: Strehlau et al., 2020**(9)
No estimates reported

**Study 10: Eales et al., 2020**(10)
No estimates reported. Only p-values presented

**Study 11: Prado et al., 2017**(11)					
	**Ghana**		**Malawi**		-
	**Language**	**Motor**	**Language**	**Motor**	-
	**Mean difference (SE), p-value**	**Mean difference (SE), p-value**	**Mean difference (SE), p-value**	**Mean difference (SE), p-value**	-
Exclusive Breastfeeding First 6 months	-0.03 (0.06), 0.65	-0.04 (0.07), 0.51	0.14 (0.23), 0.56	-0.07 (0.22), 0.74	-

**Study 12: Donald et al., 2019**(12)

	Global developmental delay				-
	Total	Girls	Boys		-
	Unadjusted odds ratio (95% CI)	Unadjusted odds ratio (95% CI)	Unadjusted odds ratio (95% CI)		-
Exclusive Breastfeeding First 6 months	1.26 (0.69; 2.29)	0.89 (0.33; 2.42)	1.59 (0.74; 3.41)		-
**Study 13: Chaudhury et al., 2021**(13)					
	**Cognitive**	**Gross motor**	**Fine motor**	**Expressive language**	**Receptive language**
Ever vs never breastfed	1.48 (0.99 to 2.22)	0.98 (0.66 to 1.46)	0.94 (0.61 to 1.46)	0.74 (0.52 to 1.05)	0.97 (0.66 to 1.44)
**Study 14: Ekholuenetale et al., 2020**(14)					
	**Optimal cognitve development**	**sub-optimal cognitve development**	**P-value**	**-**	-
**Duration of breastfeeding**					
< 12 months	600 (62.8%)	356 (37.2%)	0.761	-	-
12 months +	512 (62.1%)	313 (37.9%)		-	-
**Study 15: Namazzi et al., 2020**(15)					
	**Neurodevelopmental disability**				
	**Unadjusted odds ratio (95% CI)**	**-**	**-**	**-**	**-**
**Exclusive breastfeeding**					
Yes	1.00	-	-	-	-
Mixed feeding	1.2 (0.63 - 2.49)	-	-	-	-
**Study 16: White et al., 2020**(16)					
	**Neurodevelopmental outcomes**				
	**Unadjusted risk ratio (95% CI)**	-	-	-	-
Exclusive breastfeeding at 12 weeks	1.67 (0.74, 3.77)	-	-	-	-
Exclusive breastfeeding at 12 weeks (infants exposed to food-insecure conditions)	0.41 (0.19, 0.87)	-	-	-	-
**Study 17: Sirajee et al., 2021**(17)					
	**Below average developmental score**	**Above average developmental score**	**P-value**	-	-
Breastfed within 1 hour	64 (79)	68 (76)	0.96	-	-
**Exclusive breast feeding**					
6 weeks	72 (89)	78 (88)	0.99	-	-
12 months	0	2 (2.2)	0.5	-	-

*Sex, size for gestational age, delivery gestational age, and weight-for-age at assessment
†Child age at cognitive assessment and parent study treatment regimen.

Table S 5: Summary of subgroup analysis on the association between breastfeeding and cognitive development in sub-Saharan Africa

Odds ratios (95% Confidence interval)

Study 1: Rochat et al., 2016(1)*

	Sequential		Planning		Learning		Simultaneous		Riddle	
	**Unadjusted odds ratio (95% CI)**	**Adjusted odds ratio (95% CI)**	**Unadjusted odds ratio (95% CI)**	**Adjusted odds ratio (95% CI)**	**Unadjusted odds ratio (95% CI)**	**Adjusted odds ratio (95% CI)**	**Unadjusted odds ratio (95% CI)**	**Adjusted odds ratio (95% CI)**	**Unadjusted odds ratio (95% CI)**	**Adjusted odds ratio (95% CI)**
**Exclusive breastfeeding (Girls)**										
0-1 months	-	1.00	-	1.00	-	1.00	-	1.00	-	1.00
2-5months	-	1.34 (0.6 - 2.8)	-	0.70 (0.3 - 1.5)	-	0.57 (0.3 - 1.2)	-	1.10 (0.5 - 2.3)	-	1.00 (0.5 - 2.2)
6 months	-	1.69 (0.8 - 3.5)	-	0.49 (0.2 - 1.0)	-	0.77 (0.4 - 1.6)	-	1.07 (0.5 - 2.2)	-	0.90 (0.4 - 2.0)
**Exclusive breastfeeding (Boys)**										
0-1 months	-	1.00	-	1.00	-	1.00	-	1.00	-	1.00
2-5months	-	1.24 (0.6 - 2.5)	-	1.08 (0.5 - 2.2)	-	2.07 (1.0 - 4.3)	-	1.52 (0.7 - 3.2)	-	1.12 (0.5 - 2.4)
6 months	-	0.90 (0.5 - 1.7)	-	1.13 (0.6 - 2.1)	-	1.87 (0.9 - 3.7)	-	1.59 (0.8 - 3.1)	-	1.58 (0.8 - 3.1)

Mean difference (95% Confidence interval)

Study 3: Tumwine et al., 2018(3)†

	General cognition		Working memory		Attention		Inhibition		Cognitive flexibility	
	**Unadjusted mean difference (95% CI)**	**Adjusted mean difference (95% CI)**	**Unadjusted mean difference (95% CI)**	**Adjusted mean difference (95% CI)**	**Unadjusted mean difference (95% CI)**	**Adjusted mean difference (95% CI)**	**Unadjusted mean difference (95% CI)**	**Adjusted mean difference (95% CI)**	**Unadjusted mean difference (95% CI)**	**Adjusted mean difference (95% CI)**
Exclusive breastfeeding at 12 weeks	0.07 (-0.11 - 0.24)	0.14 (-0.12 - 0.41)	0.04 (-0.15 - 0.23)	0.07 (-0.19 - 0.31)	0 (-0.13 - 0.13)	0.06 (-0.17 - 0.29)	0.03 (-0.15 - 0.20)	0.24 (0.02 - 0.46)	-0.02 (-0.15 - 0.09)	0.05 (-0.29 - 0.19)

* Adjusted for child sex, child age, mother's age at birth, maternal IQ, mother's education at birth, birthweight, birth order, mother's HIV status, residence, income provider, owning fridge, perception of wealth, crèche, HOME assessment score, maternal mental health, and parenting stress.

† Adjusted for socioeconomic status, electricity in-home, duration in kindergarten and cluster

Table S 6: Estimates from sex-stratified subgroup analysis of the association between breastfeeding and educational achievement in sub-Saharan Africa.

**Study 2: Mitchell et al., 2015**(19)*				
	**Grade repetition**			
	**Unadjusted odds ratio (95% CI)**	**Adjusted odds ratio (95% CI)**	**-**	**-**
**Exclusive breastfeeding for six months (Girls; n = 429)**				
0-1 month	1.00	1.00	-	-
2-5 months	0.90 (0.44; 1.82)	0.98 (0.43; 2.25)	-	-
6 months	0.75 (0.39; 1.44)	0.76 (0.33; 1.74)	-	-
**Exclusive breastfeeding for six months (Boys; n = 413)**				
0-1 month	1.00	1.00	-	-
2-5 months	0.76 (0.39; 1.48)	0.67 (0.33; 1.36)	-	-
6 months	0.72 (0.39; 1.32)	0.56 (0.29; 1.10)	-	-

* Adjusted for maternal age, maternal education, residence, main income, and fridge ownership (all measured at birth) and child age, child sex, birth order, birth weight, HIV exposure

References

1. Rochat TJ, Houle B, Stein A, Coovadia H, Coutsoudis A, Desmond C, et al. Exclusive Breastfeeding and Cognition, Executive Function, and Behavioural Disorders in Primary School-Aged Children in Rural South Africa: A Cohort Analysis. PLoS Med [Internet]. 2016 Jan 1;13(6):e1002044. Available from: http://medicine.plosjournals.org/perlserv/?request=index-html&issn=1549-1676

2. Namazzi G, Hildenwall H, Mubiri P, Hanson C, Nalwadda C, Nampijja M, et al. Prevalence and associated factors of neurodevelopmental disability among infants in eastern Uganda : a population based study. BMC Pediatr. 2019;19(379):1–10.

3. Tumwine JK, Nankabirwa V, Diallo HA, Engebretsen IMS, Ndeezi G, Bangirana P, et al. Exclusive breastfeeding promotion and neuropsychological outcomes in 5-8 year old children from Uganda and Burkina Faso: Results from the PROMISE EBF cluster randomized trial. PLoS One [Internet]. 2018 Jan 1;13(2):191001. Available from: http://search.ebscohost.com/login.aspx?direct=true&AuthType=cookie,ip,shib&db=awn&AN=29474479&site=ehost-live

4. Le Roux SM, Donald KA, Brittain K, Phillips TK, Zerbe A, Nguyen KK, et al. Neurodevelopment of breastfed HIV-exposed uninfected and HIV-unexposed children in South Africa. Aids. 2018;32(13):1781–91.

5. Madlala HP, Myer L, Malaba TR, Newell ML. Neurodevelopment of HIV-exposed uninfected children in Cape Town, South Africa. PLoS One [Internet]. 2020;15(11 November):1–20. Available from: http://dx.doi.org/10.1371/journal.pone.0242244

6. McDonald CM, Manji KP, Kupka R, Bellinger DC, Spiegelman D, Kisenge R, et al. Stunting and wasting are associated with poorer psychomotor and mental development in HIV-exposed tanzanian infants. J Nutr. 2013;143(2):204–14.

7. Boivin MJ, Maliwichi-Senganimalunje L, Ogwang LW, Kawalazira R, Sikorskii A, Familiar-Lopez I, et al. Neurodevelopmental effects of ante-partum and post-partum antiretroviral exposure in HIV-exposed and uninfected children versus HIV-unexposed and uninfected children in Uganda and Malawi: a prospective cohort study. Lancet HIV. 2019;6(8):e518–30.

8. Ngoma MS, Hunter JA, Harper JA, Church PT, Mumba S, Chandwe M, et al. Cognitive and language outcomes in HIV-uninfected infants exposed to combined antiretroviral therapy in utero and through extended breast-feeding. AIDS [Internet]. 2014;28(SUPPL. 3):S323-30. Available from: http://ovidsp.ovid.com/ovidweb.cgi?T=JS&PAGE=reference&D=med11&NEWS=N&AN=24991905

9. Strehlau R, van Aswegen T, Burke M, Kuhn L, Potterton J. A description of early neurodevelopment in a cohort of HIV-exposed uninfected children. AIDS Care - Psychol Socio-Medical Asp AIDS/HIV [Internet]. 2020;32(11):1421–8. Available from: https://doi.org/10.1080/09540121.2020.1736257

10. Eales B, Krüger E, Graham M, Van Der Linde J. Feeding and developmental outcomes of infants in a South African community. Prim Heal Care Res Dev. 2020;21:1–8.

11. Prado EL, Abbeddou S, Adu-Afarwuah S, Arimond M, Ashorn P, Ashorn U, et al. Predictors and pathways of language and motor development in four prospective cohorts of young children in Ghana, Malawi, and Burkina Faso. J Child Psychol Psychiatry Allied Discip. 2017;58(11):1264–75.

12. Donald KA, Wedderburn CJ, Barnett W, Nhapi RT, Rehman AM, Stadler JAM, et al. Risk and protective factors for child development: An observational South African birth cohort. PLoS Med. 2019;16(9):1–20.

13. Chaudhury S, Williams PL, Mayondi GK, Leidner J, Holding P, Tepper V, et al. Neurodevelopment of HIV-Exposed and HIV-Unexposed Uninfected Children at 24 Months. Pediatrics. 2021;140(4).

14. Ekholuenetale M, Barrow A, Ekholuenetale CE, Tudeme G. Impact of stunting on early childhood cognitive development in Benin : evidence from Demographic and Health Survey. Egypt Pediatr Assoc Gaz. 2020;68(31).

15. Namazzi G, Tumwine JK, Hildenwall H, Ndeezi G, Mubiri P, Hanson C, et al. Neurodevelopmental outcomes of preterm babies during infancy in Eastern Uganda: a prospective cohort study. Glob Health Action [Internet]. 2020;13(1). Available from: https://doi.org/10.1080/16549716.2020.1820714

16. White M, Feucht UD, Duffley E, Molokoane F, Durandt C, Cassol E, et al. Does in utero HIV exposure and the early nutritional environment influence infant development and immune outcomes ? Findings from a pilot study in Pretoria , South Africa. Pilot Feasibility Stud. 2020;6(192):1–20.

17. Sirajee R, Conroy AL, Namasopo S, Opoka RO, Lavoie S, Forgie S, et al. Growth Faltering and Developmental Delay in HIV-Exposed Uninfected Ugandan Infants : A Prospective Cohort Study. J Acquir Immune Defic Syndr. 2021;87(1):730–40.

18. Horta BL, Bas A, Bhargava SK, Fall CHD, Feranil A, de Kadt J, et al. Infant Feeding and School Attainment in Five Cohorts from Low- and Middle-Income Countries. PLoS One. 2013;8(8):1–9.

19. Mitchell JM, Rochat TJ, Houle B, Stein A, Newell ML, Bland RM. The effect of maternal and child early life factors on grade repetition among HIV exposed and unexposed children in rural KwaZulu-Natal, South Africa. J Dev Orig Health Dis. 2015;7(2):185–96.
